# Hair and Claw Dehydroepiandrosterone Concentrations in Newborn Puppies Spontaneously Dead within 30 Days of Age

**DOI:** 10.3390/ani12223162

**Published:** 2022-11-16

**Authors:** Jasmine Fusi, Maria Cristina Veronesi, Alberto Prandi, Tea Meloni, Massimo Faustini, Tanja Peric

**Affiliations:** 1 Department of Veterinary Medicine and Animal Sciences, Università degli Studi di Milano, Via dell’Università 6, 26900 Lodi, Italy; 2 Department of Agricultural, Food, Environmental and Animal Sciences, University of Udine, Via Sondrio, 2/a, 33100 Udine, Italy; 3 Free Practitioner, DVM, Ambulatorio Veterinario Dott.ssa Tea Meloni, Via Fabio Filzi 24/28, 25063 Gardone Val Trompia, Italy

**Keywords:** dog, dead newborn, DHEA, hair, claws

## Abstract

**Simple Summary:**

Still today, information about the canine perinatal period is lacking, even if this period has many challenges. One of the essential key systems for the correct final fetal development and for the neonatal adaptation is the hypothalamic–pituitary–adrenal axis. The final products of this axis are cortisol and dehydroepiandrosterone (DHEA). The aim of the present study was to investigate DHEA concentrations in claws and hair of dead newborn puppies, classified as premature puppies (PRE-P), stillborn puppies (STILL-P) and puppies that died from the 1st to the 30th day of life (NEON-P). Results showed higher DHEA concentrations in claws of PRE-P puppies when compared with other categories. DHEA concentrations in hair did not reveal any statistical difference. The findings of the present study confirm that higher DHEA levels are produced during intrauterine life when compared with subsequent phases, also in puppies that died. The use of claws confirms their potential for long-term hormonal studies in puppies. These results provide useful information about the canine perinatal period.

**Abstract:**

The latest intrauterine fetal developmental stage and the neonatal period represent the most challenging phases for mammalian offspring. Toward the term of pregnancy, during parturition, and after birth, the hypothalamic–pituitary–adrenal axis (HPA) is a key system regulating several physiologic processes, through the production of cortisol and dehydroepiandrosterone (DHEA). This study was aimed to assess DHEA concentrations in hair and claws of 126 spontaneously dead newborn puppies, classified as premature puppies (PRE-P), stillborn puppies (STILL-P) and puppies that died from the 1st to the 30th day of life (NEON-P). The possible influence of newborn sex, breed body size, and timing of death on DHEA concentrations in both matrices was evaluated. Claw DHEA concentrations were higher in the PRE-P group when compared to STILL-P and NEON-P puppies (*p* < 0.05), whilst no significant differences were found in hair for all the studied factors. The results confirm the hypothesis that higher amounts of DHEA are produced during the intrauterine life in dogs, also in puppies that will die soon after birth.

## 1. Introduction

In veterinary medicine, the perinatal period spans from the last intrauterine fetal developmental stage until the end of the neonatal period. This phase is full of challenges for the mammalian offspring, and remains largely unstudied in canine species. At term of pregnancy, several physiologic processes are regulated from the fetal hypothalamic–pituitary–adrenal (HPA) axis, such as the fetal lung final maturation [1], the response to stress [2], and the triggering of parturition [3], all of which are well documented in scientific literature. Even after birth, and along the neonatal period, when the newborn undergoes a variety of physiologic and metabolic changes necessary for survival and well-being, the HPA axis still plays a role in the neonatal adaptation to extra-uterine life [4,5].

In response to the adrenocorticotrophic hormone, adrenals produce cortisol and dehydroepiandrosterone (DHEA), two hormones characterized by opposite effects [6]. Although cortisol has been largely used as a biomarker of chronic stress both for humans and animals, the additional evaluation of DHEA, and the estimation of their ratio [7,8], is advisable to complete the study of the HPA axis activity. In actual fact, DHEA is indeed reported as a useful marker of HPA axis activity in humans and animals [6,9] with its anti-glucocorticoid properties, as shown in the blood of men with metabolic syndrome [10].

Although cortisol, as the endpoint of stress, has broadly been investigated in literature, up to now, less data have been available on the biologic functions of DHEA, both in human and companion animals. However, recent papers have also reported that secretion of DHEA (and the product of the sulphating process, DHEA sulfate–DHEA(S)) is sometimes elicited after an acute stressor or after the challenge with the adrenocorticotrophic hormone, in humans [6,11]. Additionally, DHEA was proved to reverse the pulmonary hypertension in infant rats [12], suggesting its possible role in human neonates affected by a similar respiratory disease.

In human fetuses and newborns, HPA activity was previously investigated through neonatal blood [13] or cord blood analysis [14], and, very recently, saliva tests were used to assess DHEA concentrations in newborns [11]. These methods, however, are flawed because they provide only a single point measurement and, concerning cord blood, the collection cannot be repeated over time.

To overcome these issues, cortisol concentrations were recently studied in hair; overall, this matrix has proven to be suitable for the detection of cortisol, both in humans [15,16] and animals [17,18], dogs included [19,20,21], evidencing the usefulness of hair as a matrix for the non-invasive, and long-term retrospective measurement of previous hormone accumulation. Beyond hair, an alternative, suitable, non-invasive specimen for retrospective measurement of hormones accumulation over a long period is represented by nails (or claws). As for hair, Ben-Khelil et al. 2011 [22] showed the usefulness of nails to measure HPA axis hormones, thanks to the ability of this matrix to accumulate endogenous substances over several weeks or months; the substances (hormones included) passively diffused from capillaries to the matrix of nails and are incorporated into the keratin along the nail bed [23].

The property of accumulating hormones during a long time-window allows to reduce the number of samplings, respecting animal welfare. Another advantage is the possibility to store samples without specific precautions, wrapped in paper envelopes and kept at room temperature until the analysis, as previously reported [20,24,25].

Both cortisol and DHEA were detected in fingernails of adult humans [22], and DHEA was measured in nails of infants [2], suggesting its potential role as a biomarker of intrauterine response to maternal stress. Similarly, the availability of non-invasive samples for fetal and neonatal biology investigation is opening the doors to a deeper knowledge about both late intrauterine and early neonatal canine physiology [20,24,25]. Moreover, the refinement of non-invasive and simple techniques of investigation is particularly valuable in canine puppies, given their small dimensions.

Lastly, hair [20,26] and nails/claws [2,20,24,25] were proved to be valuable matrices for retrospective and cumulative hormonal assessment [27], allowing the investigation of hormonal changes occurring during long-term periods, such as fetal or neonatal developmental phases. To the authors’ knowledge, data about the exact timing of the first appearance of fetal claws in dogs are lacking, but anecdotally reported to be about 30 days of pregnancy, as fully visible claws are usually detectable at this time [20]. Therefore, it is possible to assume that the analysis of hormones in claws collected at birth could provide retrospective information on the last half of pregnancy in dogs. Aside from one study about the concentrations of cortisol and DHEA(S) in the claws of live newborn puppies [24], and a previous one on hair and claw cortisol concentrations in dead newborn puppies [20], data about the concentrations of DHEA in spontaneously dead puppies are lacking.

For these reasons, with the purpose of improving knowledge about the perinatal period in dog and about the related perinatal HPA axis activity, the aims of the present study were: (a) to assess the concentrations of DHEA in hair and claws in newborn puppies spontaneously dead along the perinatal period; (b) to assess the possible influence of newborn sex, breed size, and timing of death on DHEA concentrations in hair and claws in dead puppies; (c) to assess the possible correlation between hair and claw DHEA concentrations in dead puppies.

## 2. Materials and Methods

### 2.1. Ethics

The study was approved by the Università degli Studi di Milano Ethical Committee (OPBA) with protocol OPBA_147_2019. All the owners and breeders signed a written informed consent form for the collection of hair and claws from dead puppies for research purposes.

### 2.2. Animals

The study was performed on purebred puppies spontaneously dead and without gross physical malformations. After sex recording, puppies were classified as belonging to small-sized breeds (bodyweight ≤ 10 kg), or to a merged class of medium-large-sized breeds (bodyweight >10 kg), according to maternal body weight.

Based on the timing of death, puppies were grouped as follows: (1) premature puppies (PRE-P) when puppies were delivered between 2 and 1 weeks before the expected date of whelping, calculated on the base of ultrasonographic prediction of parturition date [28]; (2) stillborn puppies (STILL-P): born-dead puppies delivered at the predicted term of pregnancy plus puppies born alive at term, but dying within 24 hours of birth; (3) puppies dead between the 1st and the 30th day after birth (NEON-P).

### 2.3. Hair and Nails Samples Collection

Soon after death, a proper amount of hair (at least 20 mg) and all claw tips were carefully sampled from each puppy. Hair was shaved closest to the skin always from the same area [20], including only the back and the dorsal portion of the neck. The claw tips of all the fingers were trimmed and pooled (at least 5 mg). Hair and pooled claw samples belonging to each newborn were separately stored in paper envelopes, at room temperature, and protected from UV rays until processing.

### 2.4. Hair and Claws DHEA Assays

Hair and claw strands were washed in 3-mL isopropanol to ensure the removal of any steroids on their surface. Hair and claws DHEA were extracted with 3-mL (min 0.12 mL/mg) of methanol and measured using a solid-phase microtiter RIA assay, as described before [24,25,29].

In brief, a 96-well microtiter plate (Optiplate, Perkin-Elmer Life Science, Boston, MA, USA) was coated with goat anti-rabbit γ-globulin serum diluted 1:1000 in 0.15 mM sodium acetate buffer, pH 9, and the plate was incubated overnight at 4 °C. The plate was then washed twice with phosphate-buffered saline (PBS), 0.05 M, pH 7.5 (RIA buffer), pH 7.4, and incubated overnight at 4 °C with 200 μL of the anti-DHEA serum diluted 1:80,000. The rabbit anti-DHEA antibody used was obtained from Analytical Antibodies (Bologna, Italy). Cross-reactivities of this antibody with other steroids are as follows: DHEA 100%, 5-Androsten-3β,17β-diol 9.2%, epiandrosterone 2.8%, 5α-Androstane-3β,17β-diol 0.6%, testosterone 0.1%, androstenedione 0.1%, DHEA sulfate 0.04%, cortisol < 0.001%. After washing the plate with RIA buffer, the standards (5–200 pg/well), the quality control extract, the test extracts, and the tracer (DHEA; Perkin-Elmer Life Sciences, Boston, MA, USA) were added, and the plate was incubated overnight at 4 °C. Bound hormone was separated from free hormone by decanting and washing the wells in RIA buffer. After addition of 200 μL of scintillation cocktail (MicroScint™-20 Perkin-Elmer Life Sciences, Boston, MA, USA), the plate was counted on a β-counter (Top-Count, Perkin-Elmer Life Sciences, Boston, MA, USA). Intra- and interassay CV were 3.7 and 11.6%, respectively. The sensitivity of the assay, calculated as the interpolated dose of the response to a concentration of zero minus the statistical error, was 12.4 pg/mL.

### 2.5. Statistical Analysis

Data regarding DHEA concentrations in hair and claws were statistically analyzed by ANOVA, to assess (a) possible differences in hair and claw concentrations of DHEA, according to newborn sex and to breed size; (b) possible differences in hair or claw concentrations of DHEA according to newborn timing of death. Separate analyses were performed in order to assess the influence of single effects alone, avoiding the possible influence of factors interaction. The possible correlation between hair and claw DHEA concentrations was assessed by Spearman’s correlation test. The significance was set at *p* < 0.05 (Stata/MP 17 for Windows, College Station, TX, USA).

## 3. Results

The number of puppies enrolled in the study was 126, belonging to many canine breeds; there were 60 (47.6%) females and 66 (52.4%) males.

According to maternal body size, 53 puppies (42.1%) were classified as belonging to small-size breeds and 73 (57.9%) to medium-large-sized breeds.

Based on the timing of death, 22 puppies (17.5%) were PRE-P, 78 (61.9%) were STILL-P, and 26 (20.6%) were NEON-P.

Data regarding hair and claw DHEA concentrations in the 126 puppies, grouped according to sex and breed body size, are summarized in Table 1.

Data about hair and claws DHEA concentrations in the 126 puppies grouped according to the timing of death are reported in Table 2.

The Spearman correlation test did not show significant correlations between DHEA concentrations in hair and claws.

## 4. Discussion

The present study showed data about DHEA concentrations in hair and claws of premature born dead puppies, stillborn puppies, and newborn puppies spontaneously dead in the first 1–30 days of age, providing further evidence of the usefulness of alternative and non-invasive matrices for perinatal hormonal studies in canine species, as previously reported [20,24,25].

Because body hair in dogs appears at 44–46 days post-coitum, with the completion being reported at 52–54 days [30], it is reasonable to assume that hair collection at birth should reflect the hormonal accumulation from the first appearance of hair until the time of delivery, i.e., the last two to three weeks of pregnancy. Some studies in humans and other animal species demonstrated that maternal corticotropin releasing hormone activates the fetal HPA axis, inducing the secretion of DHEA and, to a lesser extent, cortisol [3,31,32,33]. Consequently, DHEA measured at birth in newborn hair and claws could be considered as a marker of offspring HPA activity during the last phase of intrauterine life, under the maternal influence [2].

The most relevant result of the present study was the significant effect of the timing of death on DHEA concentrations in claws, significantly higher (*p* < 0.05) in PRE-P when compared to STILL-P or NEON-P, in accordance with a previous study on cortisol concentrations in claws of dead puppies [20]. Recognizing the causes leading to premature birth as the result of a stressor, or being stressors themselves, could support the interpretation of the present study results. Consistently, DHEA concentrations were higher in the nails of human neonates delivered by mothers affected by stressful events during pregnancy [2]. These similarities could support the hypothesis that fetal DHEA production could be related to fetal intrauterine exposure to maternal stress [2].

However, when measurement of DHEA concentrations is performed after the exposure to a stressor, results can have variable patterns: in a recent report, indeed, 60% of human newborns experienced a drop in salivary concentrations of DHEA after a stressor, whereas the remaining 40% showed an increased salivary DHEA concentration [11]. Moreover, the different matrices used for the measurement of hormones, and therefore the time window of hormone accumulation, could also influence the different results obtained in these studies.

Concerning the concentrations of DHEA in newborn dogs, a previous study [24], performed on alive puppies in which claws were serially collected from birth to 60 days of age, reported higher concentrations of DHEA(S) in claws in comparison to further samplings at 30 and 60 days of age. Although data obtained in the present study could be hardly compared with those obtained in the above-mentioned study, it is noteworthy that, in the present study, puppies’ claw DHEA concentrations in all the groups were higher than those observed in alive puppies by Fusi et al. [24]. Aside from the differences in experimental design and analysis, it could be speculated that the different findings may be attributed to the condition of alive vs. dead puppies, in which the different activation of the HPA axis, and the consequent DHEA production and accumulation in claws could represent a cause or a consequence of an acute stressful condition related to the cause of death. In fact, Fustini et al. [34] reported that in cows, overstocking could lead to acute stress, resulting in increased DHEA(S) concentrations, maybe with the protective role of antagonizing cortisol effects [6].

Another hypothesis relies on the report that in humans, the fetal steroid synthesis seems to be deviated towards DHEA rather than cortisol as early as the 8th–10th week until the 32nd–36th week of pregnancy [35]. Given that, in the present study, the highest DHEA concentrations were found in prematurely delivered newborn puppies, it may be hypothesized that claw DHEA concentrations reflect the DHEA produced and accumulated during early–mid pregnancy. The subsequent DHEA decrease could reflect what observed in human babies starting from the second half of the first year of age, with a relative quiescence of the HPA axis at this time of childhood [35]. This last hypothesis is also sustained by comparing the results of the present study with data reported by Veronesi et al. [20], in which cortisol concentrations in claws were higher in premature vs born dead puppies or puppies dead 1-30 days of age. However, at present, these hypotheses should be considered very cautiously, because of the paucity of studies performed on these new matrices in dogs, and also because of the still scarce knowledge about newborn dog physiology. Similar studies were previously performed in humans, aiming at investigating DHEA concentrations in nails of infants [2]; however, the effect of age on hormonal concentrations was not demonstrated. Similarly, [36] measured DHEA concentrations in nails collected from students aged between 18 and 24 years; however, in that same study, the effect of age was not assessed.

In the present study, other parameters, such as newborn sex and breed body size, were evaluated for their possible influence on DHEA concentrations in the claws and hair of dead newborn puppies. Concerning neonatal sex, no statistical differences were found, in agreement with a recent report on live newborn puppies [24], which reinforces the hypothesis that there is no difference in the production of this hormone in the two sexes, at least during the perinatal period. Considering the breed body size, no statistical differences were found, in agreement with findings from a similar study on the concentrations of cortisol in the nails of dead puppies [20].

A final comment regards the absence of significant differences in hair DHEA concentrations according to age at death. This result is different from data previously reported for cortisol [20], in which hair cortisol concentrations were higher in prematurely dead puppies than born-dead puppies or puppies dead at 1–30 days of age. The absence of significantly higher hair DHEA concentrations in PRE-P compared with STILL-P and NEON-P is difficult to explain, and a tentative explanation could be related to a possible different mechanism of DHEA accumulation between the coat and the claws in the last fetal stage of development, which is less efficient in the coat in comparison to the claws.

This hypothesis could also be supported by the lack of correlation between the DHEA concentrations in coat and claws, suggesting a possible different pattern of DHEA accumulation in dogs during the perinatal period.

## 5. Conclusions

This study, beside confirming the usefulness of the claws as matrix for evaluating the retrospective long-term accumulation of hormones in newborn dogs, showed that DHEA concentrations in claws of spontaneously dead puppies were significantly higher in puppies delivered prematurely compared to stillborn and to puppies that died within 30 days of age, confirming the hypothesis that higher amounts of this steroid hormone are produced during intrauterine life, and also in puppies that die soon after birth.

## Figures and Tables

**Table 1:** Data (mean ± SD) about the hair and claw DHEA concentrations in the 126 puppies, grouped according to sex and breed body size.

Hormone and Matrix	Sex		Breed Body Size	
	Females (*n* = 60)	Males (*n* = 66)	Small (*n* = 53)	Medium–Large (*n* = 73)
DHEA in hair (pg/mg)	47.9 ± 14.96	44.4 ± 13.03	44.8 ± 12.91	47.1 ± 14.82
DHEA in claws (pg/mg)	27.0 ± 12.25	26.3 ± 13.96	27.5 ± 12.59	27.7 ± 11.01

**Table 2:** Data (mean ± SD) about the hair and claw DHEA concentrations in the 126 puppies grouped ac-cording to the timing of death.

Hormone and Matrix	Premature (PRE-P) (*n* = 22)	Stillborn (STILL-P) (*n* = 78)	1–30 d (NEON-P) (*n* = 26)
DHEA in hair (pg/mg)	52.3 ± 14.23	46.3 ± 13.90	44.3 ± 15.00
DHEA in claws (pg/mg)	33.8 ± 13.15 ^a^	26.6 ± 13.20 ^b^	24.7 ± 15.80 ^b^

Within row, ^(a, b)^ *p* < 0.05.

## Data Availability

The data are available upon reasonable request to the corresponding author.
